# Benefits and Costs of Animal Virulence for Microbes

**DOI:** 10.1128/mBio.00863-19

**Published:** 2019-06-04

**Authors:** Arturo Casadevall, Liise-anne Pirofski

**Affiliations:** a Department of Molecular Microbiology and Immunology, the Johns Hopkins School of Public Health, Baltimore, Maryland, USA; b Division of Infectious Diseases, Albert Einstein College of Medicine, Bronx, New York, USA; University of Texas Health Science Center at Houston

**Keywords:** benefits, costs, microbial, virulence

## Abstract

This essay is written from the vantage point of the microbial world. While the focus of much thought in the microbial pathogenesis and infectious diseases fields has been on the impact of host-microbe interaction on the host, here we ask questions about what happens to the microbe. What are the costs and benefits for microbes of having the capacity for virulence? Our exploration of this topic leads us to conclude that virulence confers very few benefits for microbes, unless disease is necessary for microbial survival through host-to-host spread. In fact, the capacity for virulence is often fraught with risk for microbes, including host dependence and the threat of extinction. The costs of virulence may explain why, relative to their enormous numbers in nature, very few microbes are actually associated with human and animal disease.

## Abstract (precis)

This essay is written from the vantage point of the microbial world. While the focus of much thought in the microbial pathogenesis and infectious diseases fields has been on the impact of host-microbe interaction on the host, here we ask questions about what happens to the microbe.

## INTRODUCTION

Is the capacity for virulence a benefit, a debit, or neutral for microbes? This topic has received relatively little attention in studies of animal microbial pathogenesis, although it is an important area of investigation in the field of plant microbial pathogenesis (1, 2). The question of why virulence occurs is rarely posed, and when it is, it is often in the context of the relatively few pathogenic fungal species for mammals or the absence of disease-causing archaea. This focus on virulence despite the fact that the overwhelming majority of microbial species are not pathogenic and the overwhelming evidence of beneficial microbial associations is likely to reflect some residual zeitgeist from earlier times when microbes were seen mostly negatively. With regard to the fungi, the question often centers on the paucity of fungal species that cause disease in humans relative to the many species associated with disease in plants and insects. For fungi, the remarkable resistance of mammals to systemic mycoses is proposed to result from the thermal barrier resulting from higher temperatures in combination with vertebrate adaptive immunity (3). In addition, ample data now suggest that innate immune mechanisms contribute to natural resistance to fungal disease (4). Since invertebrates have innate immunity, and yet they are susceptible to fungal diseases, it may be the combination of both innate and adaptive immunity that is particularly effective in providing successful antifungal defenses. Similarly, the paucity of pathogenic species among archaea is considered perplexing (5, 6). When this topic is discussed with students and colleagues, someone will inevitably ask why more of the fungi have not adapted to higher temperatures so that they can cause disease in mammals and why have some archaea have not become pathogens. These questions in themselves presuppose that the capacity for virulence is a defining microbial characteristic, a corollary of which is that microbes benefit from the capacity for virulence. Some articles laud the capacity for a certain microbe to be a “successful pathogen” (for example, see references 7 and 8), and Mycobacterium tuberculosis has been touted as the “world’s most successful pathogen” (9) based on a high prevalence of infection despite the fact that the majority of infections do not necessarily lead to disease. The use of the word “success” in the phrase “successful pathogen” implies that the quality of being pathogenic is good for the microbe. Others have noted that for some microbes with the capacity for virulence, antimicrobial resistance comes at the cost of reduced virulence (10, 11). Here, the word “cost” can be interpreted to presuppose some benefit to reduced virulence, even though the relationship between these phenotypes is complicated and under different forms of selection (12). This underscores the need to consider the state of a microbe in a host, from whence it came, and where it may go to evaluate the cost of microbial virulence (11, 12). In this essay, we review the fascinating topic of virulence, its benefits and costs, and argue that the capacity for virulence and a pathogenic lifestyle is a risky strategy for a microbe that may lead to extinction.

## ON VIRULENCE

Any discussion of benefits and costs of microbial virulence must begin with a definition of virulence. As Bull noted in the mid-1990s; “it is not easy to define virulence in a way that pleases everyone” (13). The complexity of defining virulence is that it is a microbial property that is expressed only in a susceptible host (14). Hence, virulence is a result, the outcome of an interaction in which at least two entities participate (more if the microbiome is considered in the definition of host), rather than an independent microbial property (15). Increasing the complexity of defining virulence, multiple factors contribute to host susceptibility. We recently identified 11 factors that govern susceptibility. They are grouped in the acronym “misteaching” (*m*icrobiome, *i*mmunity, *s*ex, *t*emperature, *e*nvironment, *a*ge, *c*hance, *h*istory, *i*noculum, *n*utrition, *g*enetics) to remind readers of the foibles of attributing microbial pathogenesis to any single factor (16). Consequently, it is almost impossible to separate host susceptibility from microbial virulence; virulence is only one outcome of the interaction of susceptible hosts with microbes, and it is modified by multiple factors. Because of these complexities, there are numerous definitions of virulence in the literature, but these do not capture that (at least) two entities contribute to this property (reviewed in reference 17). The definition of virulence used in this essay was proposed as part of the damage-response framework (DRF) in 1999, namely, that virulence is the “relative capacity of a microbe to cause damage in a susceptible host”; this definition *de facto* includes host and microbial contributions (17). The qualifier “relative” is part of the definition because there are no absolute measures of virulence, so this parameter needs to be measured relative to some standard (14). For example, the virulence of a mutant strain is generally measured relative to that of its parental wild type in settings where the host is kept constant. The definitional focus on host damage makes sense because this parameter is necessary to measure virulence. Virulence is a microbial phenotype that is dependent on the availability of a susceptible host for its expression (14), and this dependence on a susceptible host means that virulence is not an independent microbial property. Consequently, any discussion of its benefits and costs for individual microbes must be linked to the state of hosts and the myriad factors that affect the state of the host, with the caveat that host-microbe interaction is modifiable by known as well as unpredictable events.

## MICROBIAL STATES

**FIG 1:**
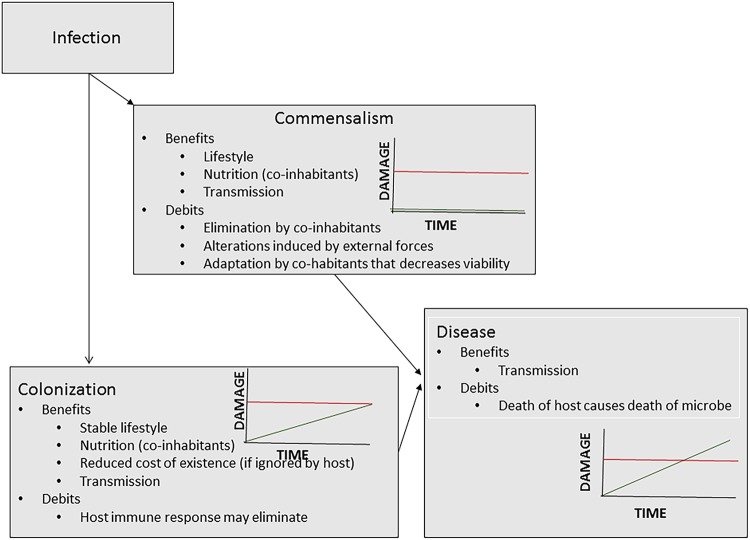
Depiction of microbial states according to the damage response framework. The possible benefits and debits for the microbe are noted for commensalism, colonization, and disease. These states, depicted for each state as a function of damage to the host (*y* axis) and time (*x* axis), differ from one another in the amount of host damage that occurs over time (green lines). The red horizontal line denotes the amount of damage when the amount of host damage translates into clinical signs and symptoms and disease occurs. Colonization and commensalism can transition to the state of disease when the amount of host damage exceeds the virulence threshold. Possible microbial benefits and debits are shown for each microbial state.

Commensalism, colonization, and disease are microbial states that are outcomes of host-microbe interaction (Fig. 1). From the perspective of the DRF (18), virulence is manifested as the state of disease, a clinical condition. For many microbes and many hosts, disease is a very uncommon outcome of host-microbe interaction. For such microbes and hosts, the more common outcome is colonization or commensalism. The host damage stemming from these states does not translate into disease. In fact, the DRF posits that commensalism, colonization, and disease are continuous states that differ only in the amounts of host damage stemming from host-microbe interaction (19). The microbe (or microbial factors (e.g., toxins), the host (e.g., inflammation), or both can cause host damage that does or does not translate into disease. However, commensalism, colonization, and disease are all states that make nutritional resources available to a microbe and can be conducive to microbial growth. However, the DRF discriminates between the host benefits and costs of host-microbe interaction. For example, no host damage is apparent in commensalism, and there may be benefits. In contrast, host damage that is sufficient to impair homeostasis results in disease, manifested as clinical illness. Although this definition implies that colonization and commensalism have no cost to and/or may benefit the host, colonization may have a cost for microbes. Host defense mechanisms can lead to microbial elimination and/or a reduction in the size of the colonizing population. Commensal populations can face the threat of elimination from interspecies competition and/or insults to the commensal (mucosal) homeostasis. Competition in microbial communities can threaten the survival of individual species. On the other hand, colonization and commensalism are states that can provide microbes a comfortable niche, including the benefit of nutrients provided by coinhabitants. In this regard, perturbations in microbial communities can increase the potential for some microbes to express their capacity for virulence (20). While the state of disease is not generally thought to benefit the individual host affected, it may benefit the microbe. Importantly, virulence is not an invariant or stable trait; thus, a microbe with the capacity for virulence in one host may not have it in another.

## THE BENEFITS OF VIRULENCE FOR MICROBES

There are three potential benefits for microbes to live in an animal host: (i) access to new sources of nutrients, (ii) transportation to new locales, and (iii) an environment that may be relatively stable. Hosts can provide a rich nutritional environment and some suggest that access to nutrients can be a selective force for virulence for certain bacteria (21). Notably, these benefits are available to microbes that live in hosts in multiple states, such as symbiosis, commensalism, and colonization. In fact, some of these states may persist for long periods and are not associated with damage that translates into disease. Hence, microbes can reap the benefits of nutrients, transport, and environmental stability without exhibiting the capacity for virulence. This is exemplified by microbial inhabitants of animal tissues in which food, transport, and environmental stability are available when the host entity is at homeostasis.

The DRF posits that the state of colonization differs from commensalism in that the host-microbe relationship results in some damage to the host that is below the threshold required for the clinical signs and symptoms that characterize the state of disease (19). Microbes in a colonizing state can benefit from nutrients, transportation, and a stable environment, provided that the damage experienced from the host immune response is not sufficient to eliminate the microbe. For example, Streptococcus pneumoniae colonization of human hosts lasts until the hosts mounts a serotype-specific immune responses that eradicates the colonizing serotype. In fact, pneumococcal vaccines are believed to work by eliciting an immune response that reduces colonization and thus the likelihood that the pneumococcus-human interaction progresses to the disease (22). Hence, the benefits of the state of colonization for the microbe may be inversely related to the amount of host damage resulting from the interaction. Nonetheless, microbes can be transmitted from a state of colonization, thus ensuring their ability to survive.

If virulence is considered a singular microbial property, it may have advantages, especially in experimental systems examining the evolution of virulence (13). A recent study featuring experimental coevolution of the host-pathogen pair Caenorhabditis elegans and Bacillus thuringiensis revealed a selective advantage for the bacterium and expression of its toxin genes (23). However, the view that virulence is an evolutionary adaptation to life in humans is problematic when one considers that some of the most virulent organisms (e.g., hemorrhagic fever virus) are zoonoses with no prior relationship with human hosts (13). On the other hand, adaptation to human hosts may lead to the perception of attenuated virulence. In reality, this may reflect the “tip of the iceberg” phenomenon, whereby the disease and its severity are most apparent in the most vulnerable hosts. When damage incurred during a host-microbe interaction is sufficient to impair homeostasis, clinical signs of symptoms of disease in the host are manifested, which if sufficiently severe produce irreversible damage and death (19).

For some microbes, there is a clear relationship between virulence and transmissibility. For example, human-to-human spread of M. tuberculosis requires aerosolization of mycobacteria by cough, which is triggered by lung damage that is largely mediated by the host inflammatory response. Similarly, for some enteric microbes, such as Vibrio cholerae and norovirus, the occurrence of disease, e.g., diarrhea, facilitates human transmission. In fact, *V. cholera* may be an extreme example of the virulence-benefit relationship, since bacterial growth in humans results in a disease that produces rice water stools in which there is a marked (several-log) amplification in the number of the bacteria, which results in a tremendous increase in infectivity (24). The same may be true of other microbes that cause diarrhea. However, for most microbes, there is no clear relationship between damage and transmissibility, especially when transmission occurs between asymptomatic hosts. For example, HIV is transmitted long before the onset of AIDS. In fact, this enhances its potential for virulence. Similarly, asymptomatic gonococcal infection in women can be efficiently transmitted to men during sexual intercourse (25). One extreme example of a microbe exploiting its capacity for virulence for survival occurs in anthrax when Bacillus anthracis grows to prodigious numbers in a dying host, sporulates, and then uses the decaying carcass to seed soils or spread to scavenging animals.

A review of human infectious diseases finds examples of host-microbe interactions where disease provides no discernible benefit to the microbe. For example, postinfectious autoimmune syndromes can occur after the infection has resolved. Neurocysticercosis results from a central nervous system inflammatory response to the pork tapeworm Taenia solium, which does not have to be alive to cause this response.

## THE COSTS OF VIRULENCE

For microbial virulence to be expressed, one needs both a microbe with pathogenic potential and a susceptible host. The pathogenic potential of a microbe is in part a function of microbial traits that result in host damage, such as production of toxins or expression of a capsule. However, the inability to express these factors does not alter microbial viability. For most microbes with pathogenic potential, virulence stems from a complex suite of factors that can be metabolically costly. The maintenance of these factors can actually reduce their growth in environments outside hosts where these factors are needed for survival (26). For example, iron acquisition requires siderophore production, which incurs significant metabolic costs (27). In the plant pathogen Ralstonia solanacearum, the microbe regulates virulence factor expression and proliferation capacity through a complex mechanism based on quorum-sensing signals (28). On the other hand, the capacity for virulence can emerge from selection forces independent of the host. For example, the virulence potential of soil fungi that are pathogenic for animals but have no need for such hosts in their life cycle is proposed to result from adaptations to an amoeboid predator that also provides a means to survive ingestion by host phagocytic cells (29). Hence, virulence can emerge accidentally as a result of traits that confer fitness in specific environments (amoeba interactions, nutrient access, climate change, etc.) that can also promote survival and host damage in a susceptible host (30). This provides further evidence that virulence may be “accidental” in the sense that microbial behavior that promotes survival in one set of circumstances may do so in one that is seemingly unrelated. Hence, it is important to avoid anthropomorphic views of virulence that invoke design or microbial preferences for a process that may arise stochastically (16).

The outcome of virulence can have significant costs for a microbe. The damage that can occur in a susceptible host may elicit immune responses that eliminate the microbe and/or confer immunity, thus eliminating that individual as a future host. Host-microbe interactions that lead to mortality for the host may select for resistant individuals and reduce the likelihood of inhabiting a susceptible host. For microbes with the capacity for virulence in humans, vaccines and therapies can eliminate or reduce opportunities for transmission to new hosts. For example, variola major poxvirus was eradicated from human populations, though it is not “extinct” because it remains in laboratory samples. Therefore, it retains the capacity for virulence, and susceptible humans enable its virulence potential. Other microbes whose host range is limited to humans, such as poliovirus and measles virus, are threatened with extinction if eradication campaigns prove successful, but only to the extent that the population remains immune. For microbes dependent on their hosts for survival, virulence carries the risk of leading the host species to extinction, especially in the setting of contributing factors, such as malnutrition or environmental stress, and/or if host populations are small and isolated. An example of this phenomenon was extinction of the native rat species on Christmas Island by a trypanosome introduced by a newly colonized rat species (31). Although the rat species extinction on Christmas Island did not lead to the extinction of the microbe, which continued to survive in other rat species, the example of host species driven to extinction by microbial virulence highlights the threat posed to some microbes.

## VIRULENCE ON BALANCE

Given the enormous numbers and diversity of microbes in the biosphere, it is remarkable how few species actually exhibit the capacity for virulence in human hosts. For example, only about 1,400 microbes, including bacteria, viruses, fungi, and parasites, have been reported as pathogenic for humans (32), and the majority of these species are rare causes of disease. This number is 9 orders of magnitude smaller than the estimated 1 trillion (10^12^) microbial species in the biosphere (33), implying that only an extremely small minority of microbes have the capacity to cause disease in humans. This number may be inflated by the fact that humans inhabit all regions of the planet and the fact that the list includes many organisms that are rarely pathogenic in intact hosts (e.g., so-called “opportunistic” pathogens, associated with conditions of impaired immunity). However, many factors restrict the opportunity for infection, e.g., only certain humans come in contact with geographically restricted microbes (e.g., such as *Coccidioides* spp., which is found only in specific regions of the Americas). On the other hand, certain factors enhance the capacity for virulence, albeit in restricted populations of humans. For example, medical care, such as surgery, radiation, chemotherapies, medications that affect the human immune response, and perturbations in the microbiome, make certain microbes more likely to exhibit virulence as an outcome of host-microbe interaction. This underscores the fact that microbial virulence is expressed only in a susceptible host. Despite the fact that microbes with the capacity for virulence include phylogenetically variable microorganisms, such as viruses and fungi, it is noteworthy that to date no members of the archaea are known to exhibit the capacity for virulence in animals. This leaves an entire domain of life out of the microbial-pathogenesis universe (5). The absence of pathogenic archaea may reflect a true limitation of this group of organisms or a sampling deficit, since we have not investigated all hosts, their microbial diseases, and their diseases of unknown etiology. Hence, it may be wise to defer judgment on the pathogenic potential of archaea until a better understanding of host-microbe relationships in the biosphere is available.

The costs and benefits of virulence are major variables driving the evolution of microbial virulence over time. A classic example of reduction in virulence over time was the experience with the rapid attenuation of myxoma virus after its introduction to control rabbits in Australia, a phenomenon that has been interpreted as an evolutionary trade-off between virulence and transmissibility (34). The evolution of microbial virulence is often viewed through the lens of microbial fitness, whereby virulence is a function of disease, host death, and transmissibility (34–36). However, virulence is not an independent microbial property because it always requires a susceptible host. Consequently, it may be worthwhile to reconsider theories of the evolution of microbial virulence from the viewpoint of host damage, rather than disease, given that damage that does not cause disease often stimulates immune responses that have detrimental consequences for microbial growth and transmissibility.

The rarity of microbes that exhibit virulence implies (i) that only rare combinations of microbial and host factors result in damage that perturbs homeostasis and/or (ii) that this potential microbial property is a costly dead end. These two explanations may not be independent since the emergence of host-microbe pairings that result in virulence can lead to the extinction of the host and possibly the microbe, thus becoming a dead end. In addition, adaptations to the host, such as genomic reduction, can reduce the host range of a microbe and make the host-microbe association exclusive, thus leaving the microbe completely dependent on a particular host. Hence, the relative rarity of microbes capable of virulence may reflect a steady state, whereby the numbers becoming extinct match those that acquire this property.

Given the variability in microbes, hosts, and outcomes of infection in different hosts at different times after infection, it is difficult, if not impossible, to do a cost-benefit calculation for microbial virulence. At first glance, the only clear benefit of virulence is when transmission of the microbe requires damage in the host. However, it is conceivable that there are unrecognized benefits to virulence, and given the complexity of host-microbe interactions, humility and nuance are probably wise stances when surveying the topic. That said, when one reviews the benefits and debits associated with a microbe having a capacity for virulence, what is most striking is how potentially costly it can be for the long-term survival of that microbial species. In fact, it is not virulence *per se* but the association with a particular host that is particularly risky, for even the benefits accrued by the states of commensalism and colonization carry the risks of host adaptation with genomic reduction and loss of capacity to inhabit other ecosystems. In this regard, the background rate of genera extinction has been estimated as 1% per million years from the fossil record, and every genus includes multiple species, a rate that has been accelerated by 3 to 4 orders of magnitude by human activity (37). Microbial species that have become totally dependent on their hosts die when the host goes extinct. Hence, host association for microbes is risky and particularly risky in the Anthropocene.
